# Ferroptosis-Mediated Formation of Tumor-Promoting Immune Microenvironment

**DOI:** 10.3389/fonc.2022.868639

**Published:** 2022-03-17

**Authors:** Qing Bi, Ze-jia Sun, Ji-yue Wu, Wei Wang

**Affiliations:** ^1^ Department of Urology, Beijing Chaoyang Hospital, Capital Medical University, Beijing, China; ^2^ Institute of Urology, Capital Medical University, Beijing, China

**Keywords:** ferroptosis, cell death, tumor microenvironment, immune escape, tumor progress, immune microenvironment

## Abstract

Ferroptosis is a newly proposed programmed cell death that has great potential in limiting tumor progression and malignancies that are resistant to conventional therapies. However, recent reports have shown that ferroptosis in the tumor microenvironment can provide a favorable environment to promote tumor survival and progression, which is induced by the infiltration and polarization of pro-tumor immune cells and the dysfunction of anti-tumor immunity. In this mini-review, we introduce the mechanisms of ferroptosis, describe the crosstalk between ferroptosis and cancer, demonstrate the potential ways in which ferroptosis shapes the pro-tumor immune microenvironment, and present our thoughts on ferroptosis-based cancer therapies.

## Introduction

Ferroptosis, which was defined by Brent R. Stockwell in 2012, is a novel form of programmed cell death driven by erastin-induced iron-dependent lipid peroxidation (LPO); moreover, it differs from apoptosis, necrosis, and autophagy (1). Further research has identified a variety of ferroptosis inducers or inhibitors (2, 3). Unlike other forms of cell death, ferroptotic cells mainly exhibit morphological changes in mitochondria, including volume reduction, condensed membrane, decreased or vanished cristae, and ruptured outer membrane (3). Iron accumulation and LPO are the main mechanisms responsible for these phenomena. Iron can either produce excess reactive oxygen species (ROS) directly through the Fenton reaction or upregulate the activity of lipoxygenase or EGLN prolyl hydroxylase, which are responsible for LPO and oxygen homeostasis. Furthermore, the inhibition of intracellular cysteine transport proteins leads to glutathione depletion, which ultimately results in the inactivation of glutathione peroxidase 4 (GPX4) and accumulation of intracellular free radicals. Excess free radicals drive the LPO of unsaturated fatty acids in the cell membrane, causing cell membrane rupture and ferroptosis. Moreover, this process can be triggered directly by GPX4 inhibitors (4, 5). Ferroptosis is widely involved in various biological processes as well as the development of several diseases, including cancer (6). The interaction between ferroptosis and cancer development, progression, and metastasis is complex; the tumor-suppressor gene *P53* can facilitate the accumulation of LPO products and thus promote ferroptosis but is broadly mutated in cancer cells (7). However, this does not necessarily indicate a downregulation of the sensitivity of tumor cells to ferroptosis. While acyl-CoA synthetase long-chain family member 4 (ACSL4) and hypoxia-inducible factor-1/2 (HIF-1/2) play important roles in cancer development, they can also upregulate the ferroptosis sensitivity (8). Moreover, ACSL4-mediated lipid metabolism has been shown to promote cancer metastasis (9). The positive role played by ferroptosis in limiting tumors and in tumor therapies has been comprehensively summarized in published reviews. However, some recent reports have indicated that ferroptosis does not play an exclusively positive role in the tumor microenvironment (TME). Therefore, in this mini-review, we aim to explore and summarize the potential mechanisms through which ferroptosis was shown to promote tumor progression by affecting the tumor immune microenvironment (TiME) in previous studies.

## Ferroptosis-Induced Infiltration and Polarization of Pro-Tumor Immune Cells

**Figure 1:**
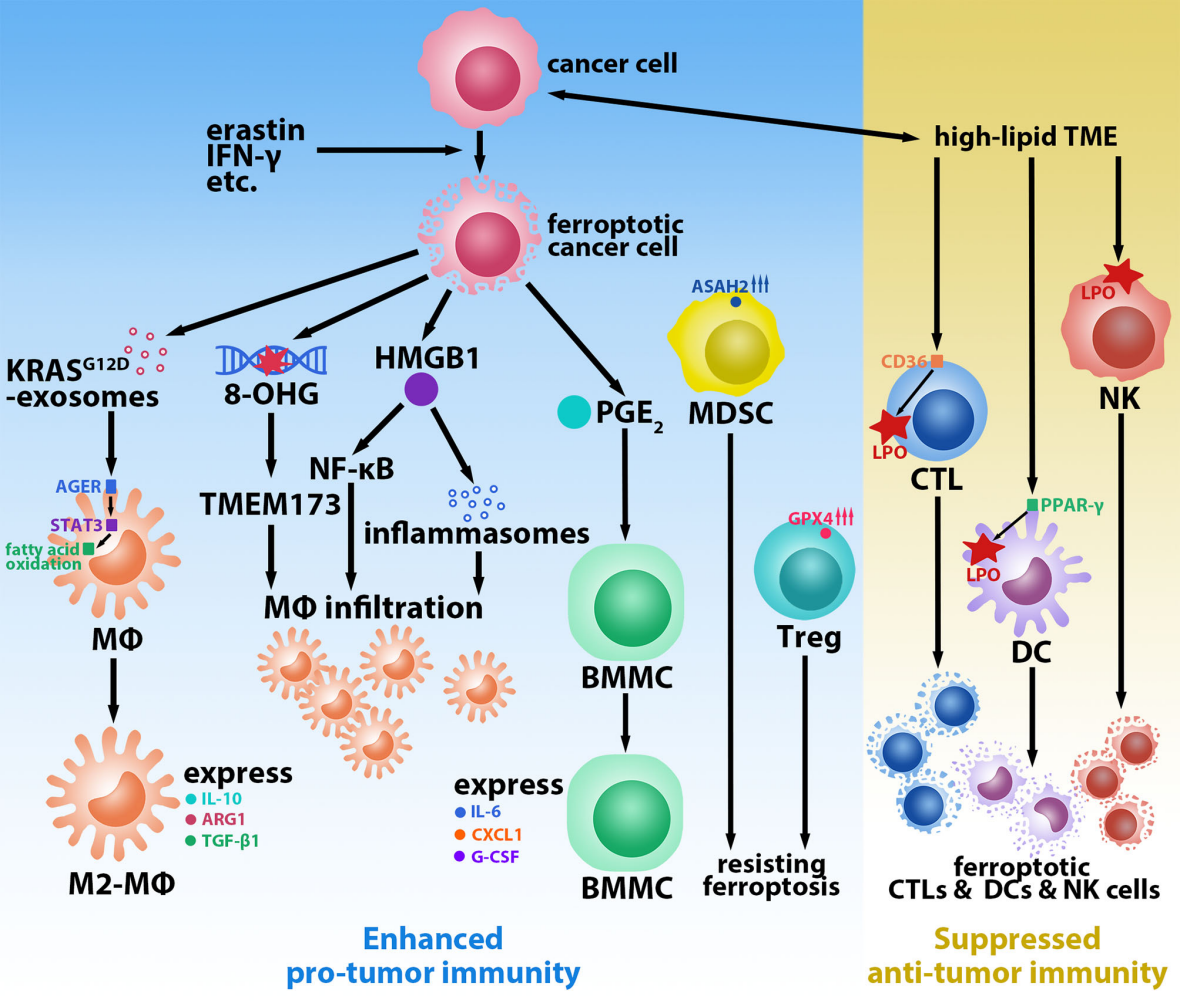
Ferroptosis-mediated tumor-promoting immune microenvironment. Ferroptotic cancer cells induced by erastin, IFN-γ, etc. promote macrophages (MΦs) infiltration and M2-polarization *via* releasing Kras^G12D^-exosomes, 8-hydroxyguanosine (8-OHG), high-mobility group box 1 (HMGB1), and prostaglandin E_2_ (PGE_2_). Regulatory T cells (Tregs) and myeloid-derived suppressor cells (MDSCs) resist ferroptosis by upregulating glutathione peroxidase 4 (GPX4) and *N*-acylsphingosine amidohydrolase (ASAH2), respectively. High-lipid conditions in the tumor microenvironment (TME) induce ferroptosis of cytotoxic T lymphocytes (CTLs) *via* CD36, natural killer (NK) cells, and dendritic cells (DCs) *via* peroxisome proliferative activated receptor-γ (PPAR-γ) by promoting lipid peroxidation (LPO).

The induction of tumor cell death, which reduces the tumor burden, is a key element in current ferroptosis-based tumor treatment strategies. However, new evidence suggests that tumor cells undergoing ferroptosis may induce a tumor-promoting TiME that leads to tumorigenesis and progression. For instance, Dai et al. found that inducing ferroptosis (via a high-iron diet or *Gpx4*-depletion) in mice led to 8-hydroxyguanosine (8-OHG) release. Elevated 8-OHG activates TMEM173/STING-dependent DNA sensor pathway and leads to macrophage infiltration; this, in turn, promotes pancreatitis and *Kras*-driven pancreatic carcinogenesis in mice (10). Furthermore, they found that KRAS^G12D^ is released in exosomes during the ferroptosis of pancreatic cancer cells with *KRAS* ^G12D^ mutation and is uptaken by macrophages *via* advanced glycosylation end product-specific receptor (AGER). KRAS^G12D^ contributes to the M2-polarization of macrophages and stimulates tumor growth *via* STAT3-dependent fatty acid oxidation pathway (11). Moreover, in macrophages, AGER also mediates the inflammation in macrophages induced by high-mobility group box 1 (HMGB1), a damage-associated molecular pattern molecule released by ferroptotic tumor cells (12). HMGB1 can accelerate the generation of pro-tumor inflammation *via* NF-κB and inflammasome pathways (13); however, the current study suggests that it plays a dual role in tumor immunity. A reduction in tumor-infiltrating macrophages and a protective effect against pancreatic cancer were observed in mice treated with deferiprone, vitamin E, and anti-HMGB1 antibodies (14). Ferroptotic tumor cells also induce an elevated expression of *PTGS2* (15), a gene that encodes PTGS2 (also called COX-2) whose downstream product is prostaglandin E_2_ (PGE_2_). Zelenay et al. found that high levels of PGE_2_ stimulated bone marrow mononuclear cells (BMMCs) to express the M2 macrophage phenotype (IL-6, CXCL1, and G-CSF) and inhibited the expression of the M1 macrophage phenotype (TNF and IL-12) in lipopolysaccharide-treated BMMCs in melanoma mice (16). Thus, macrophages occupy a key role in the ferroptosis-mediated pro-tumor immune microenvironment (**Figure 1**). While direct evidence of ferroptotic tumor cells promoting the infiltration and polarization of regulatory T cells (Tregs) and myeloid-derived suppressor cells (MDSCs)—two other key types of immunosuppressive cells in the TME (17)—is scant, they show the resistance of ferroptosis. Tregs in tumors occur little LPO (18). Promptly upregulated GPX4 expression prevents Tregs from excessive LPO and ferroptosis upon being activated (19). Similarly, tumor-infiltrating MDSCs are protected from ferroptosis by expressing high levels of *N*-acylsphingosine amidohydrolase (ASAH2) (20). These reports may indicate that few Tregs and MDSCs in the TME undergo ferroptosis, which helps them sustain pro-tumor immunity.

## Ferroptosis-Mediated Dysfunction of Anti-Tumor Immunity

CD8^+^ T cells are one of the most critical cell populations in the TME owing to their anti-tumor effects; furthermore, they play a crucial role in all stages of tumorigenesis, including the promotion of LPO and the induction of tumor ferroptosis by IFN-γ during the immunotherapies (21). The hyperlipidemic condition of the TME obliges tumor-infiltrating CD8^+^ T cells to adapt by increasing the uptake and storage of fatty acids and cholesterol *via* the upregulation of CD36 (22). However, the overexpression of CD36 induces LPO and triggers the ferroptosis of CD8^+^ T cells, leading to a decrease in the anti-tumor effectors IFN-γ and TNF-α; this accelerates tumor progression and results in poor prognosis (22, 23). Analogous to CD8^+^ T cells, natural killer (NK) cells also play an important role in defending tumors. However, Poznanski et al. observed that tumor-associated NK cells and ovarian cancer patient ascites TME-cultured peripheral blood NK cells (with notably elevated expression of LPO and ferroptosis pathways-related proteins) displayed morphological changes consistent with ferroptosis (24). Dendritic cells (DCs), which are essential antigen-presenting cells in activating cytotoxic T lymphocytes (CTLs) for anti-tumor immunity, are also affected by LPO. DCs in tumor mice and patients were identified to have elevated lipid levels, which inhibited DCs with regard to presenting antigens and activating T cells (25–27). The LPO byproduct 4-hydroxynonenal (4-HNE) can also trigger endoplasmic reticulum stress and lead to the dysfunction of tumor-driven DCs (28). Han et al. reported that peroxisome proliferative activated receptor-γ (PPAR-γ) mediated ferroptosis in DCs by being involved in the regulation of lipid metabolism (**Figure 1**). Ferroptotic DCs lose the ability to secrete TNF and IL-6, express MHC class I, and induce IFN-γ secretion by CD8^+^ T cells, thus limiting their anti-tumor abilities (29).

In addition to ferroptosis in immune cells themselves, ferroptotic tumor cells cause a similar suppression of anti-tumor immune function. As previously mentioned, M2-like tumor association macrophages, Tregs, and MDSCs are key populations involved in the suppression of the cytotoxic functions of CD8^+^ T cells and NK cells (17). Furthermore, DAMPs such as HMGB1 have also been shown to stimulate the apoptosis of DCs or induce their conversion to the CD11c^low^ CD45RN^high^ phenotype, resulting in reduced T cell activation (30). The increased release of PGE_2_, which is a widely recognized immunomodulatory factor, can also block the recruitment and activation of CD103^+^ DCs (16); moreover, it is involved in the functional inhibition of CTLs and NK cells (31, 32). In sum, these prior findings provide abundant evidence to help us link ferroptosis and potential immune escape.

## Conclusion and Prospect

The exploration of ferroptosis has provided new therapeutic ideas for limiting tumor progression and treating traditional radiotherapy- and chemotherapy-resistant cancers. However, several problems remain regarding the application of ferroptosis-targeted therapies, which need to be addressed. As previously discussed, anti-tumor immune cells in the TME are also highly sensitive to ferroptosis. Similarly, GPX4 is protective of T cells (33) and B cells (34). Therefore, inducing ferroptosis in the TME to restrict tumors will inevitably cause the death of anti-tumor immune cells, thus leading to potential immune escape. More research is needed to identify relatively specific molecules or ligands expressed by ferroptosis-sensitive tumor cells and antibody-modified nanoparticles targeting these tumor cells or tumor-specific ferroptosis pathways, which may be helpful to avoid the influence of anti-tumor immunity in ferroptosis-targeted tumor therapies. Furthermore, ferroptotic tumor cells-mediated infiltration and polarization of pro-tumor immune cells, particularly M2 macrophage populations, should not be neglected as well. Whether Tregs and MDSCs are also recruited during the process of tumor ferroptosis requires further research. Ferroptosis inducers should not be used without careful consideration and the recipients should be rigorously screened, especially as ferroptosis-inducing therapies may cause further harm to pancreatic cancer patients. In addition, while CD8^+^ T cells promote tumor ferroptosis during immunotherapy *via* IFN-γ, evidence also shows that IFN-γ can upregulate PD-L1 on the surface of cancer cells and promote tumor growth (35). As per this premise, ferroptosis-targeted therapies combined with immune checkpoint inhibitors may lead to better efficacy. It is undeniable that ferroptosis-inducing drugs not only alleviate the tumor burden but also accelerate the development of resistance. The aforementioned problems may be partially responsible for the development of drug resistance; nevertheless, this field also requires extensive further research.

## Author Contributions

QB conceived and designed the manuscript. QB wrote the original draft and designed the figure. ZS and JW reviewed and edited the draft. WW supervised and finalized the manuscript. All authors contributed to the article and approved the submitted version.

## Conflict of Interest

The authors declare that the research was conducted in the absence of any commercial or financial relationships that could be construed as a potential conflict of interest.

## Publisher’s Note

All claims expressed in this article are solely those of the authors and do not necessarily represent those of their affiliated organizations, or those of the publisher, the editors and the reviewers. Any product that may be evaluated in this article, or claim that may be made by its manufacturer, is not guaranteed or endorsed by the publisher.
